# Supplementary material for: Folic acid retention evaluation in preparations with wheat flour and corn submitted to different cooking methods by HPLC/DAD
Source: PLoS One. 2020 Apr 8;15(4):e0230583. doi: 10.1371/journal.pone.0230583 (PMC7141679; doi:10.1371/journal.pone.0230583)
Supplement: Attachment — Submitted filename: Report for Editor ,PONE-D-19-26781.docx [file pone.0230583.s001.docx]

Dear Editor

Thank you very much for considering me to review your paper PONE-D-19-26781

This paper considered the determination of folic acid in the laboratory fortified wheat and corn flour, raw preparations and cooked preparations using HPLC analysis.

In general, I recommend rejection of publication for the following reasons

The aim of the study was somewhat done in different previous studies as mentioned in the ‎references list by the authors ‎, so the novelty ‎ is so limited.

The presentation of the results is not acceptable because of ‎

- Using different units for folic acid in different tables and figures.
- Content of folic acid in the raw preparations in table 1 is different than in table 3 for ‎example for Bread Loaf is 431 ± 0,4(μg.100g-1) ± SD in table 1 and is 0,43 ± 0,030 (g/100g) ‎‎± DP even 431μg equal 0.000431‬g. ‎
- Repeated data on the tables and figures, figures 3, 4 derived from table 3.
- The folic acid peak in figure 2 is not strong peak for trusted quantification of folic acid as ‎the other peaks in the chromatogram are at so near or at the same height.
- I highly ‎recommended the authors to prepare a standard curve for folic acid inside each raw ‎preparation. After that, the authors can use this standard curve for quantification of folic acid ‎inside the raw and cooked preparations.‎

The interpretation of discussion needs more supporting results for discussing the variable ‎results ‎against the different conditions of cooked preparation of temperature and ‎foodstuff added per each ‎preparation.

Best regards, ‎‎
