# Supplementary material for: Folic acid retention evaluation in preparations with wheat flour and corn submitted to different cooking methods by HPLC/DAD
Source: PLoS One. 2020 Apr 8;15(4):e0230583. doi: 10.1371/journal.pone.0230583 (PMC7141679; doi:10.1371/journal.pone.0230583)
Supplement: Attachment — Submitted filename: Report for authors, PONE-D-19-26781.docx [file pone.0230583.s002.docx]

**Dear authors**

Please find the following suggestions and I hope to enhance your paper for good publication

**Introduction**

1- Please highlight the important of folic acid for the human health and the negative effect of its deficiency to spotlight the important of your work
2- Please highlight‎ some of the previous reports discussed thermal effect on the retention of folic acid and other micronutrients through the different cooking methods

**Material and methods**

1. **Samples and reagents**
1- Lines (88-90): Commercially fortified wheat and corn flours and laboratory fortified wheat and corn flours were evaluated in the laboratory. (what the added value of this sentence please delete it)
2- ‎Lines (91-92): ‎ different brands of wheat flour (WF); different brands of corn flour (CF) (please add the name of used brands).
3- Lines (95): ‎item 2.2. (there is no 2.2. please indicate it as 2)
2. **Protocol for laboratory folic acid fortification of wheat and corn flour**
1- Lines (108): ‎a concentration of 1000 μg of folic acid for every 100 g of wheat or corn flour. (Why you exceeded the Brazilian recommended dose; a maximum limit (220 μg.100g-1) for addition of the vitamin in order to avoid exposure of the population to an unnecessary risk (BRAZIL, 2017).
2- ‎Lines (109-110): ‎two procedures of homogenization were used (I recommended the first procedure only because it is the simplest and applicable one either for individual or industrial use, so please delete the second procedure).
3- Lines (124-127): ‎At the end of the fortification procedure, the total time used for the homogenization of the vitamin into the farinaceous matrix, including manual (1 min for each 100 g) and mechanical homogenization (1 min for every 100 g + 2 minutes for 500 g), was of 12 minutes. (Why you mentioned this sentence to compare the spent time and what about the multiple steps and tools is it ok for industrial application??)
4- Lines (128): ‎packages to prevent degradation (please describe the full conditions (temp. container material and color, humidity, etc...) you used to prevent degradation ‎ for storage until used).
3. **Food preparations**
1- Lines (133): ‎laboratory folic acid fortified wheat flour and corn flour, (which one you used? Homogenized with procedure 1 or 2).

**4.1. Sample extraction**

1- Lines (200-201): ‎The methodological procedure was a solid-liquid extraction developed and used for plant matrices by Paiva et al. (PAIVA et al., 2012). (Please replace it to; The folic acid was extracted from the samples (fortified wheat flour and corn flour, raw preparations, and cooked preparations) following the methods of PAIVA et al. (2012).‎

2- Lines (205): ‎ in 5000 g rotation (please replace in with at)

**4.2. Chromatographic conditions**

1- Please indicate the full description of HPLC equipment used in this analysis with the country of made, the type of the detector used and wavelength at which the folic acid was detected. Also, indicate the type and specification of the used column.
2- Line (218); The column was washed with water and conditioned with acetonitrile (Merck-grade HPLC) at the end of the analyses (PAIVA et al., 2012). (Please delete this sentence and move the reference in the right place)

4.3.Identification and quantification

1- (1000 μg.mL-1 folic acid) (please replace it with (1000 μg.mL^-1^ folic acid)

**5.True retention**

(MURPHY; CRINER, GRAY, 1975) (Please correct it to Murphy‎ et al., 1975)

**Results and discussion**

1- **‎**Line (256-257);‎The methodology applied allowed for there to be retention of folic acid in 18.2 minutes at maximum absorptivity in 290nm. (Please delete it because you mentioned it in the methodology section).
2- Lines (265-266);‎ 1. Determination of folic acid in commercially fortified and laboratory fortified wheat and corn flours‎ (please delete it)
3- ‎ Line (268); ‎Fig. 1 Chromatogram o commercially fortified wheat flour and corn flour. (Please make it: HPLC chromatogram of commercially fortified wheat flour and corn flour evaluated in July (A) and September (B)).
4- ‎ Lines (269-274);‎ ‎ Chromatograms for one of the samples of commercially fortified wheat flour and corn flour evaluated in July (A) and one of the wheat flour and corn flour samples evaluated in September (B) Chromatographic conditions: C18 (150 x 4.6 mm 5μm) chromatographic column (Allcrom Phenomenex) with the oven at 40 °C, isocratic elution using potassium phosphate buffer (pH 2.0) and methanol (85:15) as mobile phase at a flow of 0.5 mL/min-1 and UV detection with wavelength of 290 nm. (Please delete it and mentioned it in the methodology section‎ as general condition for all HPLC analysis), and apply it for the other figures.
5- Lines (276);‎ (1.000μg.100g-1) ( Please correct it to ‎1000μg.100g^-1‎^) and check it through the paper
6- ‎ the presentation of the results is not acceptable because of:

1- Using different units for folic acid in different tables and figures

2- Content of folic acid in the raw preparations in table 1 is different than in table 3 for example for Bread Loaf is 431 ± 0,4(μg.100g-1) ± SD in table 1 and is 0,43 ± 0,030 (g/100g) ± DP even 431μg equal 0.000431‬g and what is the meaning of DP.

3- Repeated data on the tables and figures, figures 3, 4 derived from table 3, so table 3 is enough

4- Table 1 contains extra statistical data have no effect on the study

5- Cooking conditions columns in Table 3 did not give any information as it as the same mentioned in materials and methods and are not variable studied conditions

7- The folic acid peak in figure 2 is not strong peak for trusted quantification of folic acid as the other peaks in the chromatogram are at so near or at the same height. (I highly recommended the preparation of standard curve for folic acid inside each raw preparation. After that, you can use this standard curve for quantification of folic acid inside the raw and cooked preparations.
8- Using of standard curve for folic acid inside each raw preparation will verify the retention time for folic acid on the chromatogram and will give you the recovery percent of the solvent used for folic acid extraction from the preparation and it will answer you about the big variation on the folic acid quantities on your different preparations as the extraction method has a strong effect on the recovery content of folic acid.
9- The interpretation of discussion needs more supporting results for discussing the variable ‎results ‎against the different conditions of cooked preparation of temperature and ‎foodstuff added per each ‎preparation. ‎
