# Supplementary material for: Folic acid retention evaluation in preparations with wheat flour and corn submitted to different cooking methods by HPLC/DAD
Source: PLoS One. 2020 Apr 8;15(4):e0230583. doi: 10.1371/journal.pone.0230583 (PMC7141679; doi:10.1371/journal.pone.0230583)
Supplement: Attachment — Submitted filename: Response to Reviewers.docx [file pone.0230583.s008.docx]

**Dear editorial below I send as replies of the comments and suggestions of the reviewers. For each comment below follows an answer. We appreciate all the considerations you have made and we seek the ultmost from all that has been requested.**

Reviewers' comments:

Reviewer's Responses to Questions

**Comments to the Author**

1. If the authors have adequately addressed your comments raised in a previous round of review and you feel that this manuscript is now acceptable for publication, you may indicate that here to bypass the “Comments to the Author” section, enter your conflict of interest statement in the “Confidential to Editor” section, and submit your "Accept" recommendation.

Reviewer #3: All comments have been addressed

Reviewer #7: All comments have been addressed

Reviewer #8: All comments have been addressed

Answer: Thank you for your considerations.

2. Is the manuscript technically sound, and do the data support the conclusions?

The manuscript must describe a technically sound piece of scientific research with data that supports the conclusions. Experiments must have been conducted rigorously, with appropriate controls, replication, and sample sizes. The conclusions must be drawn appropriately based on the data presented.

Reviewer #3: Partly

Reviewer #7: Partly

Reviewer #8: Yes

Answer: Thank you for your considerations.

3. Has the statistical analysis been performed appropriately and rigorously?

Reviewer #3: I Don't Know

Reviewer #7: I Don't Know

Reviewer #8: Yes

Answer: Thank you for your considerations.

4. Have the authors made all data underlying the findings in their manuscript fully available?

The [PLOS Data policy](http://www.plosone.org/static/policies.action#sharing) requires authors to make all data underlying the findings described in their manuscript fully available without restriction, with rare exception (please refer to the Data Availability Statement in the manuscript PDF file). The data should be provided as part of the manuscript or its supporting information, or deposited to a public repository. For example, in addition to summary statistics, the data points behind means, medians and variance measures should be available. If there are restrictions on publicly sharing data—e.g. participant privacy or use of data from a third party—those must be specified.

Reviewer #3: Yes

Reviewer #7: No

Reviewer #8: Yes

 Answer: Thank you for your considerations.

5. Is the manuscript presented in an intelligible fashion and written in standard English?

PLOS ONE does not copyedit accepted manuscripts, so the language in submitted articles must be clear, correct, and unambiguous. Any typographical or grammatical errors should be corrected at revision, so please note any specific errors here.

Reviewer #3: Yes

Reviewer #7: Yes

Reviewer #8: Yes

 Answer: Thank you for your considerations.

6. Review Comments to the Author

Please use the space provided to explain your answers to the questions above. You may also include additional comments for the author, including concerns about dual publication, research ethics, or publication ethics. (Please upload your review as an attachment if it exceeds 20,000 characters)

Reviewer #3: the authors have addressed my comments and explained how they collected their data but the type of column and its specifications used in HPLC analysis weren't mentioned in the chromatographic conditions (3.2) and were deleted in page12 so it has to be added.

Reviewer #7: (No Response)

Reviewer #8: The search is good, its language is intact. Statistical analysis is good. The idea is good.
But there are a few linguistic mistakes. And some references do not match the text or vice versa. Good job in the end.

Answer: References have been revised to meet the journal's standards. Please check the manuscript with markings.

7. PLOS authors have the option to publish the peer review history of their article ([what does this mean?](https://journals.plos.org/plosone/s/editorial-and-peer-review-process#loc-peer-review-history)). If published, this will include your full peer review and any attached files.


If you choose “no”, your identity will remain anonymous but your review may still be made public.


**Do you want your identity to be public for this peer review?** For information about this choice, including consent withdrawal, please see our [Privacy Policy](https://www.plos.org/privacy-policy).

Reviewer #3: No

Reviewer #7: No

Reviewer #8: Yes: Sabry AbdElMonem AbdAllah
